# Supplementary material for: Antibiotic Combination to Effectively Postpone or Inhibit the In Vitro Induction and Selection of Levofloxacin-Resistant Mutants in Elizabethkingia anophelis
Source: Int J Mol Sci. 2024 Feb 12;25(4):2215. doi: 10.3390/ijms25042215 (PMC10889675; doi:10.3390/ijms25042215)
Supplement: Supplementary file 1 [file ijms-25-02215-s001.zip › ijms-2868865-supplementary.pdf]

Supplemental Figure S1. Phylogenetic tree showing the four isolates belong to different clones, among 27 clones of *E. anophelis*

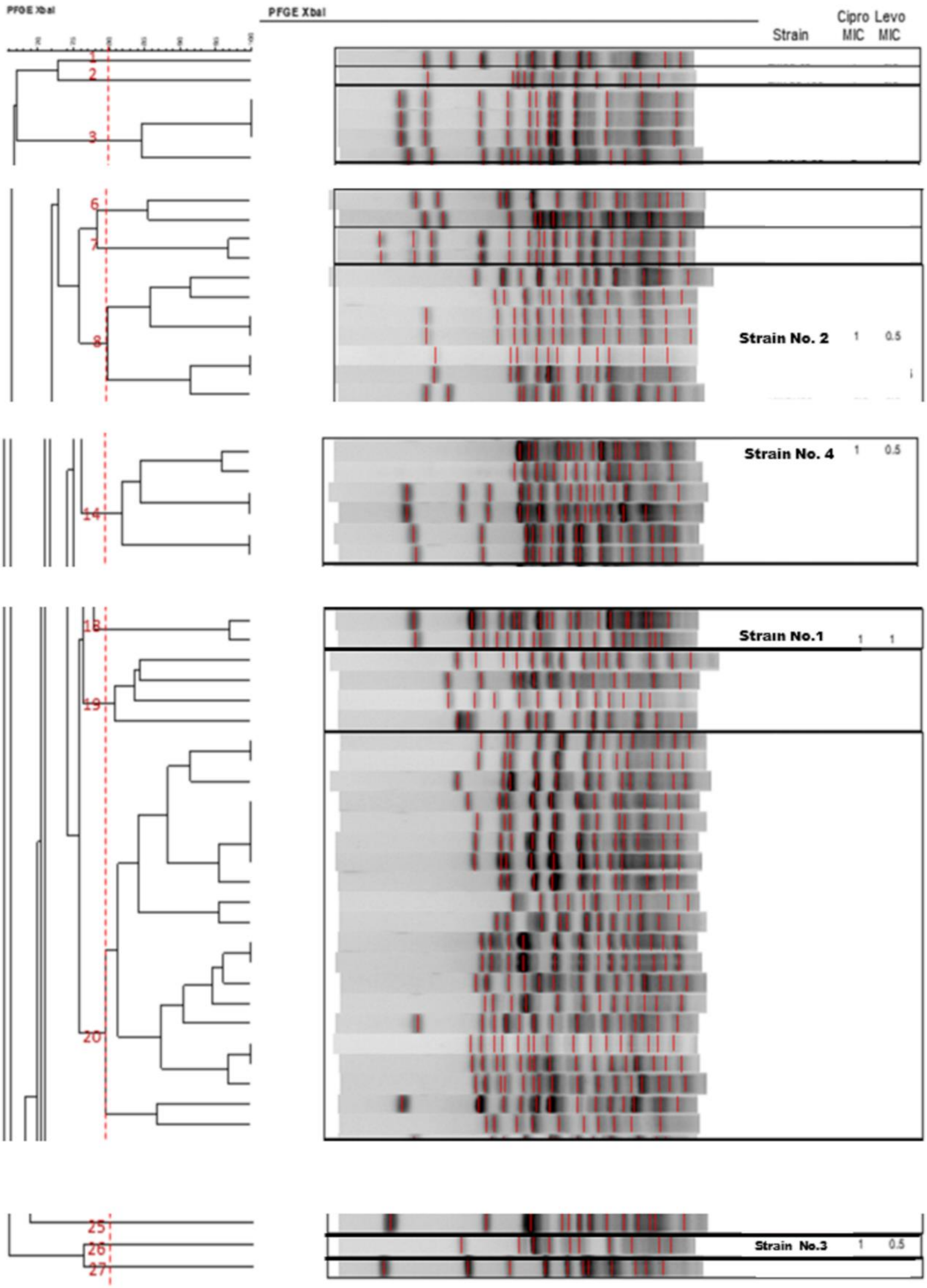

Supplemental Figure S2. Levofloxacin MICs for levofloxacin alone and its combination in each induction cycle

susceptible | intermediate | resistant

Levofloxacin	Levofloxacin MIC (mg/L) of mutant in steps							
Strain No.	0	1	2	3	4	5	6	7
1	1	4	64	512				
2	0.5	64	256	256	512			
3	0.5	32	256	512				
4	0.5	8	128	128	128	512		

Levofloxacin+0.5x MIC Mino	Levofloxacin MIC (mg/L) of mutant in steps							
Strain No.	0	1	2	3	4	5	6	7
1	0.5	4	64	128	512			
2	1	2	2	2	4	4	4	4
3	1	2	2	4	512			
4	1	1	1	4	4	32	32	32

Levofloxacin +1x MIC Mino	Levofloxacin MIC (mg/L) of mutant in steps							
Strain No.	0	1	2	3	4	5	6	7
1	0.5	4	8	8	8	64	64	64
2	1	2	2	2	4	4	4	4
3	1	2	2	2	2	2	2	1
4	1	1	1	2	2	2	2	2

Levofloxacin + 0.5x MIC Rif	Levofloxacin MIC (mg/L) of mutant in steps							
Strain No.	0	1	2	3	4	5	6	7
1	0.5	4	8	8	8	8	8	8
2	1	2	2	2	4	4	4	4
3	1	2	2	2	2	2	2	2
4	1	1	1	2	2	2	2	2

Levofloxacin + 1x MIC Rif	Levofloxacin MIC (mg/L) of mutant in steps							
Strain No.	0	1	2	3	4	5	6	7
1	0.5	4	8	8	8	8	8	8
2	1	2	5	2	4	4	4	4
3	1	2	5	2	2	2	2	2
4	1	1	1	2	2	2	2	2

Levofloxacin + 0.5 x MIC Cef/Sul	Levofloxacin MIC (mg/L) of mutant in steps							
Strain No.	0	1	2	3	4	5	6	7
1	0.5	4	8	8	8	8	8	3
2	1	4	8	8	16	16	16	16
3	1	2	2	2	2	2	2	2
4	1	1	1	2	2	2	2	2

Levofloxacin + 1 x MIC Cef/Sul	Levofloxacin MIC (mg/L) of mutant in steps							
Strain No.	0	1	2	3	4	5	6	7
1	0.5	4	8	8	8	8	8	8
2	1	2	2	2	4	8	8	8
3	1	2	2	2	2	2	2	2
4	1	1	1	2	2	2	2	2

Levofloxacin + 0.5 x MIC Sul/Tri	Levofloxacin MIC (mg/L) of mutant in steps							
Strain No.	0	1	2	3	4	5	6	7
1	0.5	2	8	8	8	8	8	8
2	1	4	4	4	4	8	8	8
3	1	2	2	8	128	128	512	512
4	1	1	1	2	2	2	2	2

Levofloxacin + 1 x MIC Sul/Tri	Levofloxacin MIC (mg/L) of mutant in steps							
Strain No.	0	1	2	3	4	5	6	7
1	0.5	2	8	8	8	8	8	8
2	1	4	4	4	4	8	8	8
3	1	2	2	2	2	2	2	2
4	1	1	1	2	2	2	2	2

Cef/Sul = cefoperazone/sulbactam; Mino = minocycline; MIC = minimum inhibitory concentration; Rif = rifampin; Sul/Tri = sulfamethoxazole/trimethoprim.

Supplemental Figure S3. The MICs of antimicrobials in combination with levofloxacin in each induction cycle.

Levofloxacin + 0.5 X MIC Mino	Minocycline MIC (mg/L) of mutant in steps							
Strain No.	0	1	2	3	4	5	6	7
1	0.25	0.5	1	1	1			
2	0.5	0.5	0.5	0.5	0.5	0.5	0.5	0.5
3	0.25	0.5	0.5	2	2	2		
4	0.25	0.25	0.5	1	1	2	2	2

Levofloxacin + 1 x MIC Mino	Minocycline MIC (mg/L) of mutant in steps							
Strain No.	0	1	2	3	4	5	6	7
1	0.25	0.5	1	1	1	1	1	1
2	0.5	0.5	0.5	0.5	0.5	0.5	0.5	0.5
3	0.25	0.5	0.5	0.5	0.5	0.5	0.5	0.5
4	0.25	0.25	0.25	0.25	0.25	0.25	0.25	0.25

Levofloxacin + 0.5 x MIC Rif	Rifamipine MIC (mg/L) of mutant in steps							
Strain No.	0	1	2	3	4	5	6	7
1	0.25	1	1	1	1	1	1	1
2	0.5	0.5	0.5	0.5	0.5	0.5	0.5	0.5
3	0.5	0.5	0.5	1	1	1	1	1
4	0.25	0.25	0.25	0.5	0.5	0.5	0.5	0.5

Levofloxacin + 1 x MIC Rif	Rifamipine MIC (mg/L) of mutant in steps							
Strain No.	0	1	2	3	4	5	6	7
1	0.25	1	1	1	1	1	1	1
2	0.5	0.5	0.5	0.5	0.5	0.5	0.5	0.5
3	0.5	0.5	0.5	1	1	1	1	1
4	0.25	0.25	0.25	0.5	0.5	0.5	0.5	0.5

Levofloxacin + 0.5 x MIC Cef/Sul	Brosym MIC (mg/L) of mutant in steps							
Strain No.	0	1	2	3	4	5	6	7
1	16	128	128	128	128	128	128	128
2	256	256	256	256	256	256	256	256
3	256	256	256	256	256	256	256	256
4	256	256	256	256	256	256	256	256

Levofloxacin + 1 x MIC Cef/Sul	Brosym MIC (mg/L) of mutant in steps							
Strain No.	0	1	2	3	4	5	6	7
1	16	128	128	128	128	128	128	128
2	256	256	256	256	256	256	256	256
3	256	256	256	256	256	256	256	256
4	256	256	256	256	256	256	256	256

Levofloxacin + 0.5 x MIC Sul/Tri	SXT MIC (mg/L) of mutant in steps							
Strain No.	0	1	2	3	4	5	6	7
1	16	>32	>32	>32	>32	>32	>32	>32
2	8	>32	>32	>32	>32	>32	>32	>32
3	2	>32	>32	>32	>32	>32	>32	>32
4	4	>32	>32	>32	>32	>32	>32	>32

Levofloxacin + 1 x MIC Sul/Tri	SXT MIC (mg/L) of mutant in steps							
Strain No.	0	1	2	3	4	5	6	7
1	16	>32	>32	>32	>32	>32	>32	>32
2	8	>32	>32	>32	>32	>32	>32	>32
3	2	>32	>32	>32	>32	>32	>32	>32
4	4	>32	>32	>32	>32	>32	>32	>32

Cef/Sul = cefoperazone/sulbactam; Mino = minocycline; MIC = minimum inhibitory concentration; Rif = rifampin; Sul/Tri = sulfamethoxazole/trimethoprim.

Supplemental Table S1. The minimum inhibitory concentrations (MICs) of levofloxacin against *E. anopheles* and point mutation in quinolone resistance-determining regions in each step of multicycle induction and mutant selection by levofloxacin plus rifampin (0.5× and

Levofloxacin +1× MIC Rifampin					Levofloxacin +0.5× MIC Rifampin				
Strain	Cycle	Levofloxacin MIC (mg/L)	Point mutation in Gyr A (*gyrA*)	Point mutation in GyrB (*gyrB*)	Strains	Cycle	levofloxacin MIC (mg/L)	Point mutation in Gyr A (*gyrA*)	Point mutation in GyrB (*gyrB*)
No. 1	0	0.5	N/D	N/D	No. 1	0	0.5	N/D	N/D
	1	4	N/D	N/D		1	4	N/D	N/D
	2	8	N/D	N/D		2	8	N/D	N/D
	3	8	N/D	N/D		3	8	N/D	N/D
	4	8	N/D	N/D		4	8	N/D	N/D
	5	8	N/D	N/D		5	8	N/D	N/D
	6	8	N/D	N/D		6	8	N/D	N/D
	7	8	N/D	N/D		7	8	N/D	N/D
No. 2	0	1	N/D	N/D	No. 2	0	1	N/D	N/D
	1	2	N/D	N/D		1	2	N/D	N/D
	2	2	N/D	N/D		2	2	N/D	N/D
	3	2	N/D	N/D		3	2	N/D	N/D
	4	4	N/D	N/D		4	4	N/D	N/D
	5	4	N/D	N/D		5	4	N/D	N/D
	6	4	N/D	N/D		6	4	N/D	N/D
	7	4	N/D	N/D		7	4	N/D	N/D
No. 3	0	1	N/D	N/D	No. 3	0	1	N/D	N/D
	1	2	N/D	N/D		1	2	N/D	N/D

	2	2	N/D	N/D		2	2	N/D	N/D
	3	2	N/D	N/D		3	2	N/D	N/D
	4	2	N/D	N/D		4	2	N/D	N/D
	5	2	N/D	N/D		5	2	N/D	N/D
	6	2	N/D	N/D		6	2	N/D	N/D
	7	2	N/D	N/D		7	2	N/D	N/D
No. 4	0	1	N/D	N/D	No. 4	0	1	N/D	N/D
	1	1	N/D	N/D		1	1	N/D	N/D
	2	1	N/D	N/D		2	1	N/D	N/D
	3	2	N/D	N/D		3	2	N/D	N/D
	4	2	N/D	N/D		4	2	N/D	N/D
	5	2	N/D	N/D		5	2	N/D	N/D
	6	2	N/D	N/D		6	2	N/D	N/D
	7	2	N/D	N/D		7	2	N/D	N/D

Supplemental Table S2. The minimum inhibitory concentrations (MICs) of levofloxacin against *E. anophelis* and point mutation in quinolone resistance-determining regions in each step of multicycle induction and mutant selection by levofloxacin plus cefoperazone/sulbactam (0.5× and 1×MIC)

Levofloxacin + 1× MIC cefoperazone/sulbactam					Levofloxacin + 0.5× MIC cefoperazone/sulbactam				
Strain	Cycle	Levofloxacin MIC (mg/L)	Point mutation in Gyr A (*gyrA*)	Point mutation in GyrB (*gyrB*)	Strain	Cycle	Levofloxacin MIC (mg/L)	Point mutation in Gyr A (*gyrA*)	Point mutation in GyrB (*gyrB*)
No. 1	0	0.5	N/D	N/D	No. 1	0	0.5	N/D	N/D
	1	4	N/D	N/D		1	4	N/D	N/D
	2	8	N/D	N/D		2	8	N/D	N/D
	3	8	N/D	N/D		3	8	N/D	N/D
	4	8	N/D	N/D		4	8	N/D	N/D
	5	8	N/D	N/D		5	8	N/D	N/D
	6	8	N/D	N/D		6	8	N/D	N/D
	7	8	N/D	N/D		7	8	N/D	N/D
No. 2	0	1	N/D	N/D	No. 2	0	1	N/D	N/D
	1	2	N/D	N/D		1	4	N/D	N/D
	2	2	N/D	N/D		2	8	N/D	N/D
	3	2	N/D	N/D		3	8	N/D	N/D
	4	4	N/D	N/D		4	16	N/D	N/D
	5	8	N/D	N/D		5	16	N/D	N/D
	6	8	N/D	N/D		6	16	N/D	N/D
	7	8	N/D	N/D		7	16	N/D	N/D
No. 3	0	1	N/D	N/D	No. 3	0	1	N/D	N/D

	1	2	N/D	N/D		1	2	N/D	N/D
	2	2	N/D	N/D		2	2	N/D	N/D
	3	2	N/D	N/D		3	2	N/D	N/D
	4	2	N/D	N/D		4	2	N/D	N/D
	5	2	N/D	N/D		5	2	N/D	N/D
	6	2	N/D	N/D		6	2	N/D	N/D
	7	2	N/D	N/D		7	2	N/D	N/D
No. 4	0	1	N/D	N/D	No. 4	0	1	N/D	N/D
	1	1	N/D	N/D		1	1	N/D	N/D
	2	1	N/D	N/D		2	1	N/D	N/D
	3	2	N/D	N/D		3	2	N/D	N/D
	4	2	N/D	N/D		4	2	N/D	N/D
	5	2	N/D	N/D		5	2	N/D	N/D
	6	2	N/D	N/D		6	2	N/D	N/D
	7	2	N/D	N/D		7	2	N/D	N/D
